# Number needed to treat with ursodeoxycholic acid therapy to prevent liver transplantation or death in primary biliary cholangitis

**DOI:** 10.1136/gutjnl-2019-319057

**Published:** 2019-12-16

**Authors:** Maren H Harms, Rozanne C de Veer, Willem J Lammers, Christophe Corpechot, Douglas Thorburn, Harry L A Janssen, Keith D Lindor, Palak J Trivedi, Gideon M Hirschfield, Albert Pares, Annarosa Floreani, Marlyn J Mayo, Pietro Invernizzi, Pier Maria Battezzati, Frederik Nevens, Cyriel Y Ponsioen, Andrew L Mason, Kris V Kowdley, Bettina E Hansen, Henk R van Buuren, Adriaan J van der Meer

**Affiliations:** 1 Department of Gastroenterology and Hepatology, Erasmus University Medical Center, Rotterdam, The Netherlands; 2 Service d'Hépatologie, Centre de Référence des Maladies Inflammatoires des Voies Biliaires, Hôpital Saint-Antoine, APHP, Paris, France; 3 The Sheila Sherlock Liver Centre and UCL Institute for Liver and Digestive Health, Royal Free Hospital, London, UK; 4 Toronto Centre for Liver Disease, Francis Family Liver Clinic, Toronto Western Hospital Liver Centre, Toronto, Ontario, Canada; 5 College of Health Solutions, Arizona State University, Phoenix, Arizona, USA; 6 Department of Gastroenterology and Hepatology, Mayo Clinic, Rochester, Minnesota, USA; 7 Birmingham NIHR Biomedical Research Centre and Centre for Liver Research, University of Birmingham, Birmingham, UK; 8 Liver Unit, Hospital Clínic, CIBERehd, IDIBAPS, University of Barcelona, Barcelona, Spain; 9 Department of Surgery, Oncology and Gastroenterology, Universita degli Studi di Padova, Padova, Italy; 10 Digestive and Liver Diseases, UT Southwestern Medical Center, Dallas, Texas, USA; 11 Division of Gastroenterology and Program for Autoimmune Liver Diseases, International Center for Digestive Health, Department of Medicine and Surgery, University of Milan-Bicocca, Monza, Italy; 12 Department of Health Sciences, University of Milan, Milano, Italy; 13 Department of Hepatology, UZ Leuven, Leuven, Belgium; 14 Department of Gastroenterology and Hepatology - location Academic Medical Center, Amsterdam University Medical Centres, Amsterdam, The Netherlands; 15 Division of Gastroenterology and Hepatology, University of Alberta, Edmonton, Alberta, Canada; 16 Liver Care Network and Organ Care Research, Swedish Medical Center, Seattle, Washington, USA

**Keywords:** primary biliary cirrhosis, clinical decision making, hepatobiliary disease, liver

## Abstract

**Objective:**

The clinical benefit of ursodeoxycholic acid (UDCA) in primary biliary cholangitis (PBC) has never been reported in absolute measures. The aim of this study was to assess the number needed to treat (NNT) with UDCA to prevent liver transplantation (LT) or death among patients with PBC.

**Methods:**

The NNT was calculated based on the untreated LT-free survival and HR of UDCA with respect to LT or death as derived from inverse probability of treatment weighting-adjusted Cox proportional hazard analyses within the Global PBC Study Group database.

**Results:**

We included 3902 patients with a median follow-up of 7.8 (4.1–12.1) years. The overall HR of UDCA was 0.46 (95% CI 0.40 to 0.52) and the 5-year LT-free survival without UDCA was 81% (95% CI 79 to 82). The NNT to prevent one LT or death within 5 years (NNT_5y_) was 11 (95% CI 9 to 13). Although the HR of UDCA was similar for patients with and without cirrhosis (0.33 vs 0.31), the NNT_5y_ was 4 (95% CI 3 to 5) and 20 (95% CI 14 to 34), respectively. Among patients with low alkaline phosphatase (ALP) (≤2× the upper limit of normal (ULN)), intermediate ALP (2–4× ULN) and high ALP (>4× ULN), the NNT_5y_ to prevent one LT or death was 26 (95% CI 15 to 70), 11 (95% CI 8 to 17) and 5 (95% CI 4 to 8), respectively.

**Conclusion:**

The absolute clinical efficacy of UDCA with respect to LT or death varied with baseline prognostic characteristics, but was high throughout. These findings strongly emphasise the incentive to promptly initiate UDCA treatment in all patients with PBC and may improve patient compliance.

**Significance of this study**

**What is already known on this subject?**

- Ursodeoxycholic acid is a safe drug that is recommended for the treatment of patients with primary biliary cholangitis (PBC).
- Ursodeoxycholic acid treatment has been associated with a reduced relative risk of liver transplantation or death.
- Up to 30% of patients with PBC is currently not being treated with ursodeoxycholic acid in Western cohorts.

**What are the new findings?**

- Although the relative risk reduction related to ursodeoxycholic acid treatment with respect to liver transplantation or death is relatively stable over patients’ baseline characteristics, the absolute clinical efficacy varies but is generally high.
- The clinical efficacy of ursodeoxycholic acid can be estimated individually according to patients’ GLOBE score.

**How might it impact on clinical practice in the foreseeable future?**

- Based on these findings, physicians should initiate treatment with ursodeoxycholic acid in all patients suffering from PBC.
- Patients may be more willing to start treatment and remain compliant based on their individually calculated clinical efficacy measure.

## Introduction

Primary biliary cholangitis (PBC) is a chronic disease of the liver characterised by destruction of the small intrahepatic bile ducts and formation of hepatic fibrosis.^1 2^ It was recently estimated that nowadays 40% of patients with PBC will develop cirrhosis within 10 years, at which point patients are at increased risk of liver failure and hepatocellular carcinoma.^3^ As a result, the overall survival of patients with PBC is substantially impaired as compared with that of a matched general population.^4^

The choleretic and hydrophilic bile acid ursodeoxycholic acid (UDCA) is currently considered as the standard of care for patients with PBC.^5–7^ Based on long-term clinical experience, UDCA is considered to have a favourable safety profile. The strong association between UDCA therapy and prolonged liver transplantation (LT)-free survival was recently substantiated in both a large American cohort and our own international cohort, with a dose–response relationship highlighting the importance of the 13–15 mg/kg dose recommendation.^8^ Still, even in recent Western cohorts, as much as 30% of patients remained untreated and suboptimal UDCA dosages were frequently used.^9 10^ More awareness of and attention for the clinical efficacy of UDCA are thus needed in order to optimise the medical management and clinical outcome of the population with PBC.

While previous studies only assessed the relative reduction of the risk of clinical outcomes with UDCA therapy, our understanding of the impact of UDCA could benefit from reports of absolute measures of clinical efficacy. The number needed to treat (NNT) to prevent one clinical event represents such an absolute clinical efficacy measure with clear interpretation for physicians, patients and policymakers. Currently, it is not known how many patients with PBC should be treated with UDCA to prevent one LT or death. Although previously we showed that the relative risk reduction with UDCA is stable over various patient characteristics, the absolute risk reduction may not be.^8^ In this study we aimed to assess the NNT with UDCA to prevent one LT or death among patients with PBC. The secondary aims were to evaluate the NNT in various subgroups of patients with PBC and to estimate the NNT for the individual patient with PBC.

## Patients and methods

### Study population and design

For the current study we used the data of patients included in the database of the Global PBC Study Group, which is an international collaboration between liver units across eight countries in Europe and Northern America. The database contains data from representative long-term followed cohorts on an individual patient level of both UDCA-treated and untreated patients. All patients had an established diagnosis of PBC according to internationally accepted guidelines.^6 7^ Patients were only included in case of sufficient follow-up (>6 months and ≥2 recorded visits) and when dates of starting UDCA treatment and/or clinical events were known. For the current analyses we excluded patients in case an autoimmune overlap syndrome, based on the Paris criteria,^11^ or other concomitant liver disease was present. Further details on the methodology of data collection have been described in further detail elsewhere.^8 12^ In line with our previous work, 3902 patients were included for the current analyses.^8^

### Statistical analysis

The outcome measure of the current study was the combined endpoint of LT and all-cause mortality. Baseline was considered to be the first centre visit in untreated patients and the start of treatment in patients receiving UDCA. Treatment with UDCA for PBC is recommended lifelong and usually initiated promptly after diagnosis. Patients were followed until LT or death. In patients who remained alive without LT, the follow-up was censored at their last visit to the centre. Patients were considered lost to follow-up when it was unclear whether they were either alive, deceased or underwent LT at the time of data collection. Missing baseline data were assumed to be missing at random and were handled by means of multiple imputation (SAS Proc MI, Markov Chain Monte Carlo method). Hereto, 10 databases were generated with use of Rubin’s rules to estimate the parameters and the SE. The biochemical values included for imputation were alkaline phosphatase (ALP), aspartate aminotransferase, alanine aminotransferase (ALT), total bilirubin, albumin and platelet count. Categorical or binary variables were not imputed.

Because treatment was not assigned randomly in our study population, our analyses were performed following inverse probability treatment weighting (IPTW).^13^ Hereto, following stabilisation, weights were assigned to each individual patient based on the predictive values derived from a logistic regression model, including baseline patient characteristics and laboratory parameters (age, gender, calendar year of diagnosis, total bilirubin, ALP, ALT, platelet count, albumin), with UDCA therapy as dependent variable.^8 14^ After weighting a balance assessment was performed which previously showed that there were no remaining differences in baseline characteristics between the UDCA-treated and untreated patients.^15^ Subsequently, the association between time to LT or death and UDCA therapy was assessed through Cox proportional hazard regression analyses.

The NNT to prevent one LT or death within (t) years with UDCA therapy can be calculated with the observed LT-free survival in patients without treatment and the estimated benefit of UDCA on LT-free survival, which are both derived from IPTW-adjusted Cox regression analyses. The NNTs were estimated using the following formula: NNT=(1/(LT-free survival_untreated_(t)^HR UDCA^) − (LT-free survival_untreated_(t))).^16^ The 95% CI of both the LT-free survival and the HR of UDCA was taken into account to address the uncertainty of the NNT. Unrounded numbers of HR and untreated survival were used to calculate the NNT. The NNT was always rounded up. Although the NNT to prevent one LT or death can be calculated for every time point (t) during the follow-up (NNT_(t)y_), we primarily report the NNT to prevent one LT or death within 5 years (NNT_5y_) throughout the manuscript. Stratified analyses were performed based on categorised baseline characteristics.

The individualised NNT_5y_ was estimated using the GLOBE score, a validated objective prognostic tool which accurately predicts LT-free survival after 1 year of UDCA therapy. The GLOBE score is calculated with the following formula: 0.044378 × age + 0.93982 × LN(bilirubin) + 0.335648 × LN(ALP) + 2.266708 × albumin + 0.002581 × platelets (per 10^9^/L) + 1.216865 (bilirubin and ALP in ‘× upper limit of normal’ and albumin in ‘× lower limit of normal’).^17^ First, the predictive accuracy of the GLOBE score (calculated with the variables at baseline) for LT or death was assessed in untreated patients using the c-statistic.^18 19^ Calibration analyses were performed by comparing the predicted mortality rates with those observed, stratified for four range categories of the GLOBE score. Second, a multivariable Cox regression model for LT or death including the GLOBE was constructed. Linearity was assessed by including polynomial terms, which remained included in the multivariate model in case these were statistically significantly associated with the outcome measure. Subsequently, the HR of UDCA was calculated for each value of the GLOBE score. With the GLOBE score thus representing an untreated LT-survival estimate and an HR of UDCA, the NNT could be predicted for the individual patient.

The clinical efficacy of UDCA treatment was also assessed according to biochemical response at year 1. Hereto, patients were stratified based on their ALP level, with a cut-off of 1.67× the upper limit of normal (ULN). An ALP ≥1.67× ULN was defined as a suboptimal response. Cox proportional hazard analyses provided HRs regarding LT or death of UDCA per response group and were adjusted for all baseline characteristics as these are associated with both the long-term outcome and the biochemical response to UDCA. The untreated LT-free survival was estimated based on the median GLOBE score at baseline, thus prior to initiation of UDCA therapy, in each response group.

All statistical tests were two-sided, and a p value <0.05 was considered to be statistically significant. The significance level for interactions was set at p<0.01 to correct for multiple testing. Statistical analyses were performed in SPSS Statistics V.21.0 and SAS V.9.4.

### Patient and public involvement statement

Patients were not involved in the design or conduct of this study.

## Results

### Cohort characteristics

Included in the study were 3902 patients with PBC, predominantly female (91.0%) and with a mean (SD) age of 54.3 (11.9). Treated with UDCA were 3529 (90.4%) patients and not treated with UDCA were 373 (9.6%) patients. In our study the median (IQR) interval between the first centre visit and start of UDCA was 2.9 months (0–29). Table 1 shows the baseline characteristics according to the treatment with UDCA prior to IPTW. Following adjustment with IPTW there were no remaining baseline characteristics which differed statistically significantly between the two groups. Patients were followed for a median of 7.8 (IQR 4.1–12.1) years. Of a total of 3902 patients, 306 patients (7.8%) were lost to follow-up. During follow-up, a total of 299 patients underwent LT and 567 patients died. The primary endpoint of LT or death was observed in 721 UDCA-treated patients and 145 untreated patients.

**Table 1:** Baseline characteristics

	Overall n=3902	UDCA-treated n=3529	Untreated n=373	P value
Age at diagnosis, years*	52.3 (11.9)	52.1 (11.7)	54.1 (13.4)	<0.001
Female, n (%)	3552/3902 (91.0)	3209/3529 (90.9)	343/373 (92.0)	0.510
AMA-positive, n (%)	3507/3862 (90.8)	3175/3491 (90.9)	332/371 (89.5)	0.418
Year of diagnosis†	1996 (1990–2003)	1997 (1990–2003)	1992 (1982–2000)	<0.001
Histological disease stage, n (%)‡				<0.001
Stage I	784/2173 (36.1)	739/2076 (35.6)	45/97 (46.4)	
Stage II	671/2173 (30.9)	657/2076 (31.6)	14/97 (14.4)	
Stage III	365/2173 (16.8)	351/2076 (16.9)	14/97 (14.4)	
Stage IV	353/2173 (16.2)	329/2076 (15.8)	24/97 (24.7)	
Serum bilirubin (ULN)†	0.63 (0.44–1.00)	0.62 (0.44–1.00)	0.65 (0.43–1.38)	0.081
Serum ALP (ULN)†	2.29 (1.41–3.95)	2.32 (1.46–4.00)	1.94 (1.11–3.51)	<0.001
Serum AST (ULN)†	1.53 (1.03–2.31)	1.56 (1.05–2.34)	1.25 (0.75–2.00)	<0.001
Serum ALT (ULN)†	1.68 (1.05–2.63)	1.71 (1.09–2.68)	1.20 (0.75–1.83)	<0.001
Serum albumin (LLN)†	1.15 (1.06–1.25)	1.15 (1.06–1.25)	1.15 (1.03–1.26)	0.840
Platelet count (x 10^9^/L)†	245 (190–300)	248 (195–303)	217 (146–271)	<0.001
Biochemical disease stage, n (%)§				<0.001
Early	1576/2296 (68.6)	1376/1980 (69.5)	200/316 (63.3)	
Advanced	559/2296 (24.3)	484/1980 (24.4)	75/316 (23.7)	
Severe	161/2296 (7.0)	120/1980 (6.1)	41/316 (13.0)	

Serum bilirubin was missing for 1020 (26%) patients, serum ALP for 1069 (27%), serum AST for 1175 (30%), serum ALT for 1294 (33%), serum albumin for 1533 (39%) and platelet count for 1720 (44%). AMA status was missing for 40 (1.9%) patients.

*Data are expressed as mean and SD.

†Data are expressed as median and IQR.

‡Histological disease stage according to Ludwig and Scheuer’s classification.^28^

§Biochemical disease stage according to Rotterdam criteria.^29^

ALP, alkaline phosphatase; ALT, alanine aminotransferase;AMA, antimitochondrial antibodies; AST, aspartate aminotransferase; LLN, lower limit of normal; UDCA, ursodeoxycholic acid; ULN, upper limit of normal.

### NNT_5y_ with UDCA to prevent one LT or death

**Figure 1:**
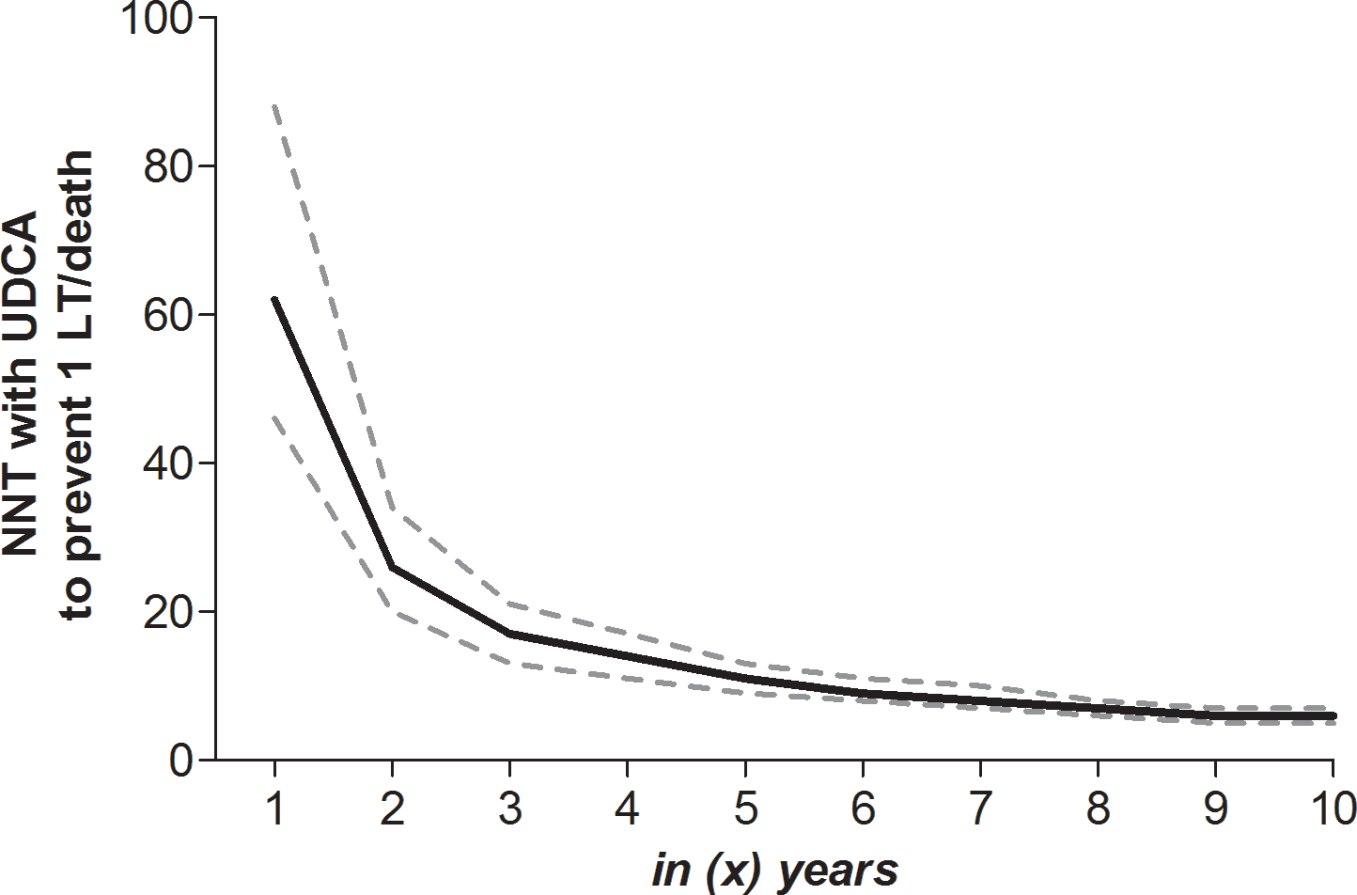
Adjusted NNT to prevent one LT or death according to treatment duration. The solid line represents the adjusted NNT with UDCA among patients with PBC to prevent one LT or death according to various treatment durations on the x-axis. The dotted lines represent the 95% CI, which are based on both the 95% CI of the adjusted HR of UDCA with respect to the occurrence of LT or death and 95% CI of the cumulative LT-free survival in patients without UDCA therapy. Results are adjusted for sex, age, year of diagnosis, albumin, platelet count, bilirubin, alkaline phosphatase, aspartate aminotransferase and alanine aminotransferase. LT, liver transplantation; NNT, number needed to treat; PBC, primary biliary cholangitis; UDCA, ursodeoxycholic acid.

Following IPTW adjustment, the 5-year cumulative LT-free survival without UDCA therapy was 81.0% (95% CI 79.3 to 82.7). The overall adjusted HR of UDCA for LT or death was 0.46 (95% CI 0.40 to 0.52, p<0.001). As a result, the NNT_5y_ to prevent LT or death in one patient was 11 (95% CI 9 to 13). With a proportional HR of UDCA over time, the cumulative LT-free survival in untreated patients at (t) years drives the estimated NNT to prevent one LT or death over that specific duration of therapy. With a 10-year cumulative LT-free survival of 60.7% (95% CI 58.2 to 63.4) in the absence of UDCA, the NNT_10y_ to prevent one LT or death was 6 (95% CI 5 to 7). Figure 1 shows the NNT_(t)y_ to prevent one LT or death according to various durations of UDCA therapy.

### Relative risk reduction versus absolute risk reduction in stratified subgroups

Table 2 presents the adjusted HRs of UDCA with respect to LT or death, the adjusted cumulative LT-free survival in UDCA-untreated patients and the adjusted NNT to prevent one LT or death within 5 and 10 years for various subgroups of patients. As previously described, the HR of UDCA for LT or death was stable over the baseline characteristics and only differed statistically significantly among patients stratified according to their baseline age and ALP and albumin levels.^8^

**Table 2:** NNT with UDCA to prevent one LT or death in 5 and 10 years in subgroups of patients with PBC

Characteristics	Adjusted HR (95% CI)*	P value HR	Untreated LT-free survival_5y_ (95% CI)	NNT_5y_ (95% CI)†¶	Untreated LT-free survival_10y_ (95% CI)	NNT_10y_ (95% CI)†¶
Sex						
Male	0.52 (0.35 to 0.77)	0.0011	0.68 (0.60 to 0.76)	8 (5 to 21)	0.55 (0.46 to 0.64)	6 (4 to 15)
Female	0.44 (0.38 to 0.52)	<0.0001	0.82 (0.80 to 0.84)	11 (9 to 14)	0.62 (0.59 to 0.64)	6 (5 to 7)
Age (years)						
≤46.0	0.33 (0.24 to 0.46)	<0.0001	0.83 (0.79 to 0.86)	9 (7 to 14)	0.60 (0.55 to 0.66)	5 (3 to 6)
46.0–62.7	0.46 (0.37 to 0.56)	<0.0001	0.80 (0.78 to 0.83)	10 (8 to 14)	0.67 (0.64 to 0.71)	7 (5 to 9)
>62.7	0.60 (0.48 to 0.76)	<0.0001	0.81 (0.77 to 0.84)	14 (9 to 28)	0.52 (0.47 to 0.58)	7 (5 to 13)
Cirrhosis‡						
No	0.32 (0.24 to 0.42)	<0.0001	0.92 (0.90 to 0.95)	20 (14 to 34)	0.71 (0.66 to 0.76)	6 (5 to 8)
Yes	0.31 (0.24 to 0.40)	<0.0001	0.48 (0.42 to 0.54)	4 (3 to 5)	0.33 (0.27 to 0.39)	3 (3 to 4)
Disease stage§						
Early	0.37 (0.30 to 0.47)	<0.0001	0.92 (0.91 to 0.94)	22 (17 to 32)	0.78 (0.75 to 0.80)	8 (6 to 11)
Intermediate	0.32 (0.25 to 0.40)	<0.0001	0.62 (0.57 to 0.67)	5 (4 to 6)	0.22 (0.17 to 0.28)	3 (3 to 4)
Advanced	0.50 (0.37 to 0.70)	0.0001	0.26 (0.20 to 0.34)	5 (3 to 8)	0.14 (0.92 to 0.20)	5 (4 to 9)
ALP						
≤2× ULN	0.61 (0.45 to 0.82)	0.0014	0.90 (0.87 to 0.92)	26 (15 to 70)	0.79 (0.75 to 0.82)	13 (8 to 35)
2–4× ULN	0.46 (0.36 to 0.59)	<0.0001	0.82 (0.79 to 0.85)	11 (8 to 17)	0.59 (0.56 to 0.64)	6 (4 to 8)
>4× ULN	0.36 (0.25 to 0.52)	<0.0001	0.66 (0.62 to 0.70)	5 (4 to 8)	0.41 (0.36 to 0.46)	4 (3 to 5)
Bilirubin						
≤ULN	0.39 (0.32 to 0.48)	<0.0001	0.91 (0.90 to 0.92)	19 (15 to 27)	0.75 (0.72 to 0.78)	7 (6 to 10)
>ULN	0.40 (0.33 to 0.48)	<0.0001	0.49 (0.45 to 0.53)	4 (4 to 5)	0.20 (0.16 to 0.25)	4 (3 to 4)
Albumin						
<LLN	0.32 (0.24 to 0.43)	<0.0001	0.35 (0.29 to 0.41)	3 (3 to 4)	0.15 (0.11 to 0.21)	3 (3 to 4)
≥LLN	0.46 (0.40 to 0.54)	<0.0001	0.87 (0.86 to 0.89)	16 (13 to 21)	0.68 (0.66 to 0.71)	7 (6 to 9)
Platelet count						
<150×10^9^/L	0.48 (0.35 to 0.46)	0.0007	0.52 (0.47 to 0.58)	5 (4 to 9)	0.27 (0.22 to 0.34)	4 (3 to 7)
≥150×10^9^/L	0.44 (0.37 to 0.52)	<0.0001	0.86 (0.84 to 0.87)	14 (11 to 18)	0.68 (0.65 to 0.70)	7 (5 to 8)

*HRs were adjusted for sex, age, year of diagnosis, albumin, platelet count, bilirubin, alkaline phosphatase, aspartate aminotransferase and alanine aminotransferase;.

†The 95% CI of the NNT was based on both the 95% CI of the adjusted HR of UDCA as well as on the 95% CI of the cumulative 5-year LT-free survival in patients without UDCA therapy.

‡Baseline histological data were available for 2173 patients;.

§Biochemical disease stage according to Rotterdam criteria.^29^

¶As the number needed to treat always needs to be rounded up, decimal differences in absolute clinical efficacy cannot be presented.

ALP, alkaline phosphatase; LLN, lower limit of normal; LT, liver transplantation; NNT, number needed to treat; PBC, primary biliary cholangitis; UDCA, ursodeoxycholic acid; ULN, upper limit of normal; 5y, 5 years; 10y, 10 years.

As example, when comparing the relative reduction of the risk of LT or death with UDCA therapy between patients with early biochemical disease (adjusted HR 0.37, 95% CI 0.30 to 0.47) or patients with intermediate biochemical disease (adjusted HR 0.32, 95% CI 0.25 to 0.40) with patients with advanced biochemical disease (adjusted HR 0.50, 95% CI 0.37 to 0.70), a small but not significant difference is observed. In absolute terms, however, the adjusted NNT_5y_ to prevent one LT or death was substantially higher among those with early biochemical disease (22, 95% CI 17 to 32) as opposed to those with intermediate or advanced disease (5 (95% CI 4 to 6) and 5 (95% CI 3 to 8), respectively). The beneficial NNT in patients with advanced biochemical response is explained by the higher 5-year cumulative incidence of LT or death (26.2%, 95% CI 20.4 to 33.7).

The IPTW-adjusted HR of UDCA was statistically significantly stronger among the youngest quartile of patients (≤46.0 years; 0.33, 95% CI 0.24 to 0.46) as compared with those in the interquartile age range (46.0–62.7 years; 0.46, 95% CI 0.37 to 0.56) and the oldest quartile of patients (>62.7 years; 0.60, 95% CI 0.48 to 0.76), while the cumulative 5-year LT-free survival rates without UDCA were rather similar among the three age groups. The stronger adjusted HR of UDCA among patients ≤46 years resulted in an adjusted NNT_5y_ to prevent one LT or death of 9 (95% CI 7 to 14), which was lower as compared with 10 (95% CI 8 to 14) in those aged 46.0–62.7 years and 14 (95% CI 9 to 28) in those older than 62.7 years.

### Predicted individual NNT to prevent one LT or death

**Figure 2:**
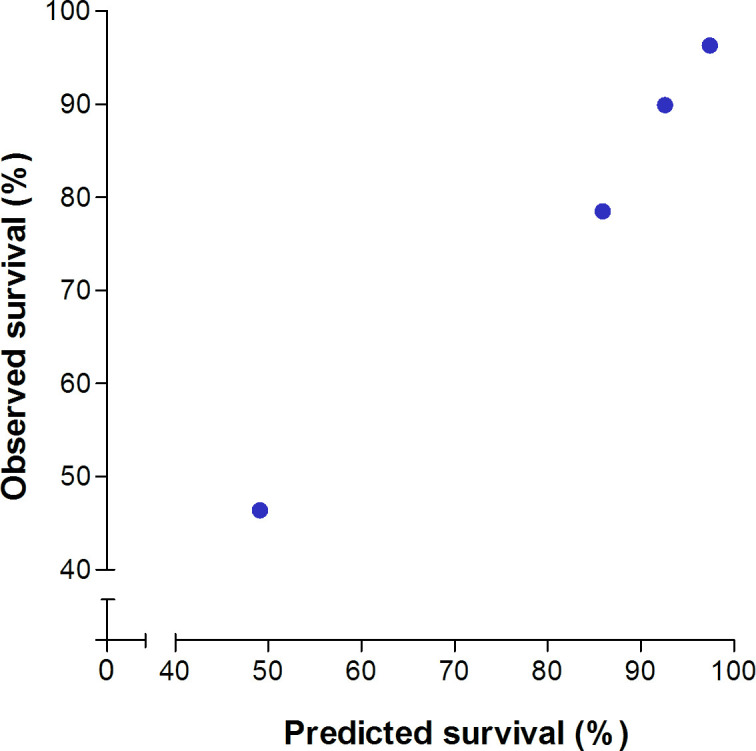
Observed versus predicted LT-free survival according to categorised risk groups. The dots represent four subgroups of patients in our cohort based on GLOBE score range with the corresponding mean predicted LT-free survival (x-axis) and observed LT-free survival (y-axis). From left to right: (1) a GLOBE score of >0.91, corresponding to a 5-year risk of >20%; (2) a GLOBE score of 0.51–0.91, corresponding to a 5-year risk of 10%–20%; (3) a GLOBE score of −0.21 to 0.51, corresponding to a 5-year risk of 5%–10%; and (4) a GLOBE score ≤−0.21, corresponding to a 5-year risk of ≤5%. LT, liver transplantation.

**Figure 3:**
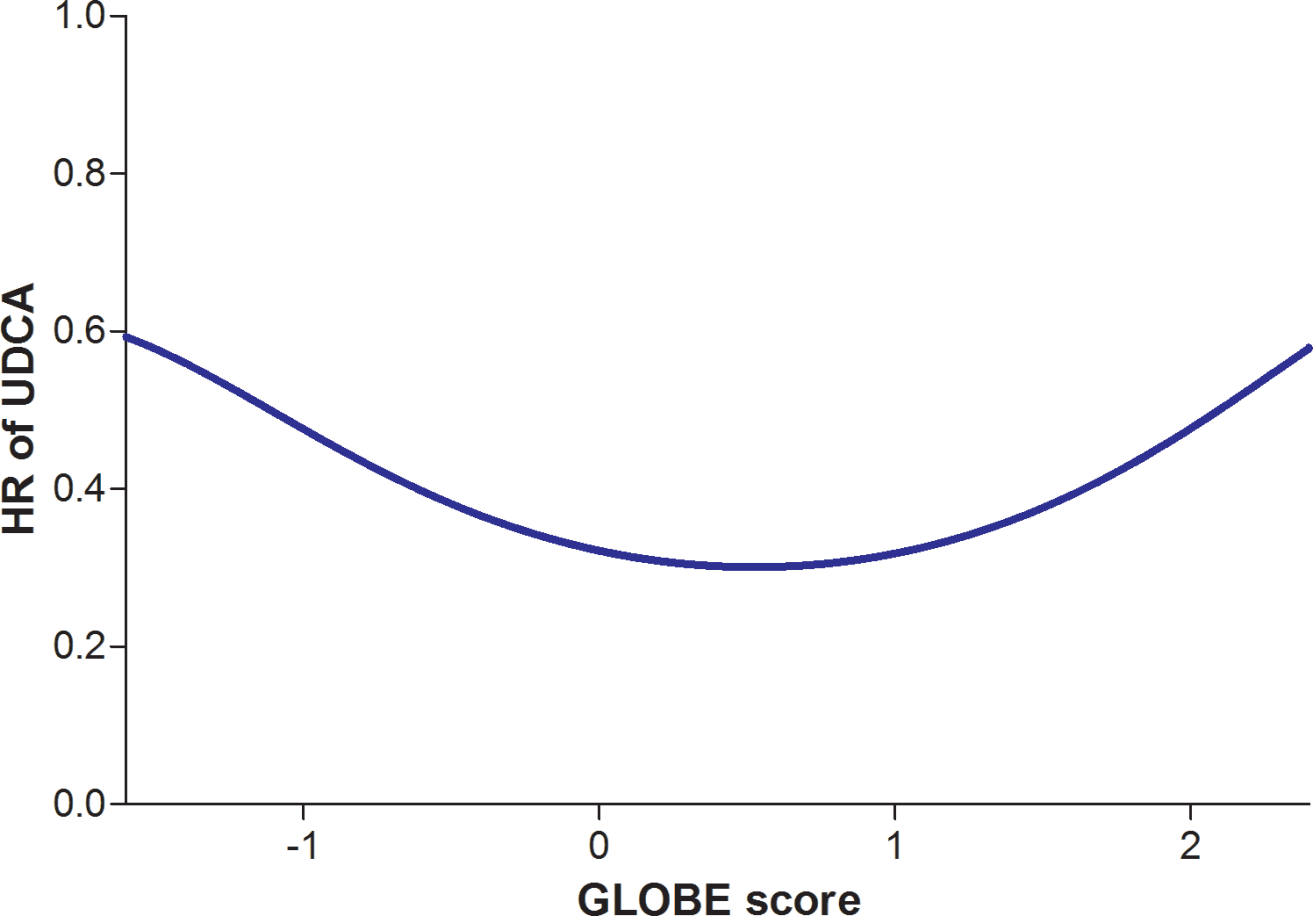
HR of UDCA on LT-free survival according to the GLOBE score. The graph shows a non-linear relationship, in which the function of the GLOBE score was significant to the fourth degree. LT, liver transplantation; UDCA, ursodeoxycholic acid.

**Figure 4:**
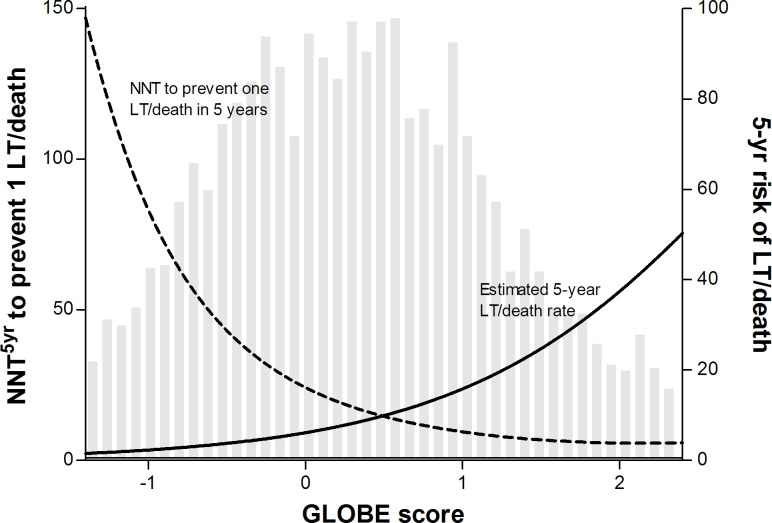
Individualised NNT_5y_ according to the GLOBE score, visualised against the estimated 5-year risk of LT or death. The solid line represents the estimated 5-year risk of LT or death according to the GLOBE score, plotted against the right y-axis. The dotted line represents NNT for 5 years to prevent the occurrence of one LT or death according to the GLOBE score, plotted against the left y-axis. The grey bars represent an independent histogram of the number of patients in our cohort according to their GLOBE score, in which the number of patients represented by the bars is shown on the left y-axis. 5yr, 5 years; LT, liver transplantation; NNT, number needed to treat.

In the untreated population, the discriminative ability of the GLOBE score was strong with a c-statistic of 0.81 (95% CI 0.78 to 0.85). The observed 5-year transplant-free survival was in line with the predicted estimates using the GLOBE score (figure 2). Figure 3 shows the polynomial function of the HR of UDCA according to the GLOBE score, which was significant to the fourth degree. Using the estimated 5-year survival that relates to every value of the GLOBE score, we predicted the NNT_5y_ for any given GLOBE score (figure 4). An NNT_5y_ ≤10 to prevent one LT/death is achieved in patients with a GLOBE score ≥0.94 (NNT_10y_=5), an NNT_5y_ of 20 in patients with a GLOBE score of 0.10 (NNT_10y_=9), while the NNT_5y_ is ≥50 in case the GLOBE score is <−0.62 (NNT_10y_=20).

### Relative risk reduction versus absolute risk reduction according to biochemical response

In our cohort, 2084 (59.1%) UDCA-treated patients had an ALP <1.67× ULN at year 1, and their 5-year and 10-year LT-free survival rates were 94.0% and 84.7%. These patients had a lower risk of LT or death (adjusted HR 0.35, 95% CI 0.29 to 0.42, p<0.0001) as opposed to those without UDCA. In contrast, 1445 (40.9%) patients had a suboptimal biochemical response. In these patients the 5-year and 10-year LT-free survival rates were 88.0% and 70.9%. Although less strong, a suboptimal response to UDCA remained associated with a statistically significantly lower risk of LT or death as compared with no UDCA (adjusted HR 0.42, 95% CI 0.36 to 0.50, p<0.0001).

Among patients with an ALP <1.67× ULN, the median GLOBE score prior to UDCA treatment was −0.0266, which translates into an estimated 5-year and 10-year LT-free survival rates of 94.0% and 84.7%. As a result, the NNT was 26 (95% CI 24 to 29) and 11 (95% CI 10 to 12) to prevent one LT or death in 5 or 10 years, respectively. In contrast, the median GLOBE score prior to UDCA treatment was 0.6978 among patients with a suboptimal biochemical response, leading to estimated 5-year and 10-year LT-free survival rates of 88.0% and 70.9%. As a result, their NNT_5y_ was 15 (95% CI 14 to 18) and the NNT_10y_ was 7 (95% CI 6 to 8).

## Discussion

In our large international cohort, the overall number of patients with PBC which needed to be treated with UDCA to prevent one LT or death within 5 years was 11, as the related relative risk reduction was 2.2 and the cumulative 5-year incidence of LT/death in untreated patients was approximately 19%. This NNT, as an absolute measure of clinical efficacy, further decreased in case one LT/death had to be prevented over longer periods of time. This is relevant, as UDCA is recommended as a lifelong therapy for patients with PBC. The NNT fluctuated according to baseline patient characteristics, which is predominantly explained by differences in the natural history of PBC in various subgroups. Nevertheless, the clinical efficacy of UDCA in terms of the NNT to postpone one LT or death with at least 5 years can be considered low throughout.

In the current study, the NNT was assessed across all relevant patient subgroups. We previously found that the relative reduction in the risk of LT/death associated with UDCA was generally stable.^8^ For instance, the HR of UDCA was similar among patients with cirrhosis (HR 0.33) and patients without cirrhosis (HR 0.31). However, the absolute clinical efficacy of UDCA was considerably lower among patients with cirrhosis (NNT_5y_=4) as compared with those without cirrhosis (NNT_5y_=20). This difference is explained by the substantially higher cumulative 5-year incidence of LT/death in untreated patients with cirrhosis (52%) than in those without cirrhosis (7%). This emphasises the relevance of appreciating the clinical setting when evaluating the clinical benefit of a therapeutic intervention, which is considered when using the NNT as a measure of efficacy. The relative risk reduction associated with UDCA with respect to LT/death did differ according to ALP, age and albumin.^8^ ALP is an established prognostic marker for long-term outcome.^12 20^ Among patients with a high ALP level (>4× ULN), the HR of UDCA was stronger and the cumulative 5-year incidence of LT/death in the absence of treatment was higher in comparison with patients with lower ALP levels. Both factors contributed to the considerably lower NNT_5y_ to prevent LT/death in patients with high ALP (5) than in those with low ALP levels (≤2× ULN: 26) before the initiation of UDCA. Young age was associated with a stronger relative risk reduction related to UDCA treatment. Although younger age is normally inversely associated with the risk of death, patients who develop PBC at a young age are known to have a more aggressive phenotype.^21^ Indeed, the cumulative LT-free survival among untreated patients with PBC aged ≤46 years in our cohort was similar as compared with that of older patient subgroups. As a result, the NNT_5y_ was only slightly lower in patients ≤46 years (9) as compared with patients aged 46–63 years (10) and >63 (14). In line with the above, the absolute clinical efficacy of UDCA therapy was stronger among patients with a suboptimal biochemical response at year 1, despite an inferior relative risk reduction. Although this might seem counterintuitive, this is explained by the impaired untreated LT-free survival in these patients when compared with those with an ALP <1.67× ULN after year 1.

As exemplified in the previous paragraph, the untreated prognosis strongly affects the absolute clinical efficacy of UDCA. For an individual patient, multiple baseline characteristics need to be considered, while it would be desirable to estimate a single patient-specific NNT. We showed that the GLOBE score, originally developed as an objective tool to estimate LT-free survival after 1 year on UDCA treatment, also accurately predicts prognosis in untreated patients. Hereafter, we estimated the individualised clinical efficacy of UDCA according to the GLOBE score. In this analysis we allowed the HR of UDCA to fluctuate with the GLOBE score as it incorporates ALP and age, the two variables with most profound and significant impact on the relative risk reduction of UDCA. An estimation of an individual NNT can be helpful for patient counselling and supporting therapeutic compliance. For example, patients might be more willing to accept perceived side effects due to an improved understanding of the expected absolute risk reduction. Noteworthy is that a high NNT with UDCA was usually a result of a favourable natural history rather than the absence of a relative benefit of UDCA.

To the best of our knowledge, this is the first study to assess the benefit of UDCA treatment in PBC in absolute risk reduction as measured by the NNT to prevent clinical endpoints. Assessment of the NNT is rare in the field of hepatology, but has recently gained popularity in many other fields of medicine. The advantage of the NNT is that it is easy to interpret for both patients and physicians as it combines the therapy-induced relative risk reduction and patients’ a priori risk of unfavourable outcome in a single parameter. The NNT can be expressed for any given treatment duration, which is especially relevant for chronic diseases such as PBC in which lifelong treatment is required. While policymakers may be interested in long-term effects of therapy, patients are more likely to prioritise short-term benefits. Moreover, physicians’ willingness to treat is reported to be dependent of the measure in which treatment benefit is presented. Providing information on both relative and absolute clinical efficacy may therefore prevent misinterpretation and aid well-informed decision making in daily clinical practice.^22–24^

As part of our study we validated the GLOBE score to accurately predict the LT-free survival in untreated patients with PBC. The availability of such an objective natural history score is relevant, also in light of novel second-line therapies which will no longer be compared with a placebo arm given the strong evidence for a beneficial effect of UDCA for all patients with PBC.^8^ The GLOBE score can thus aid to evaluate the potential additional benefit of new drugs that are added to the treatment with UDCA, and might be preferable over older prediction models such as the Mayo Risk Score as it is solely based on readily available and objective parameters.^25^

Strengths of the current study include the use of a large, internationally representative cohort with long-term follow-up and many clinical endpoints in both UDCA-treated and untreated patients. Furthermore, to ensure accurate estimation of the NNT, both the 95% CI of the HR of UDCA as well as the CI of the estimated survival in the untreated population were taken into account. Additionally, in a sensitivity analysis in patients diagnosed in or after 1990, performed to ensure results are compatible with present-day clinical practice, the relative risk reduction associated with UDCA therapy was similar (data not shown). A number of limitations should also be noted. First, a potential selection bias in this study is represented by the fact that the majority of included patients were treated in tertiary liver centres. Second, potential improvement in survival within the timespan that is chosen to express the NNT is not considered when using the NNT as a measure of risk reduction, which could thus lead to an underestimation of treatment benefit. Third, the NNT assumes a causal relationship between UDCA and prolonged LT-free survival. This has long been subject to debate, especially due to Cochrane reporting an absence of treatment benefit.^26^ As this is a retrospective study in which IPTW was used to adjust for the small differences in baseline characteristics, residual confounding can never be fully ruled out. We are lacking data on the reasons for not treating patients with PBC with UDCA, but especially shortly after its introduction it can be hypothesised that physicians may have been unaware of UDCA or not convinced about its benefits. Also, patients may have been unwilling to use this relatively new drug at that time. Because UDCA has no relevant contraindications, however, we consider it to be unlikely that the association between UDCA and improved LT-free survival is completely confounded by a patient-related factor which would have influenced both the chance of receiving UDCA and the risk of LT or death. In fact, both the positive association with clinical outcome in extensively adjusted analyses in large cohort studies and the finding of an improved LT-free survival in UDCA-treated patients with advanced disease in an older randomised controlled trial have provided a general consensus on the assumed causal UDCA treatment benefit.^6–8 27^

In conclusion, in this first study to assess the efficacy of UDCA in absolute measures, we report that the NNT with UDCA to prevent LT or death is generally low, but can be assessed for individual patients with PBC. These results provide a clear understanding of the clinical importance of optimised UDCA therapy for patients and doctors, thereby stimulating compliance and treatment uptake.

## References

[1] Kaplan MM, Gershwin ME Primary biliary cirrhosis. N Engl J Med Overseas Ed 2005;353:1261–73. 10.1056/NEJMra043898 16177252

[2] Carey EJ, Ali AH, Lindor KD Primary biliary cirrhosis. The Lancet 2015;386:1565–75. 10.1016/S0140-6736(15)00154-3 26364546

[3] Trivedi PJ, Lammers WJ, van Buuren HR, et al Stratification of hepatocellular carcinoma risk in primary biliary cirrhosis: a multicentre international study. Gut 2016;65:321–9. 10.1136/gutjnl-2014-308351 25567117

[4] Prince M, Chetwynd A, Newman W, et al Survival and symptom progression in a geographically based cohort of patients with primary biliary cirrhosis: follow-up for up to 28 years. Gastroenterology 2002;123:1044–51. 10.1053/gast.2002.36027 12360466

[5] Working subgroup for clinical practice guidelines for primary biliary C. guidelines for the management of primary biliary cirrhosis: the intractable hepatobiliary disease Study Group supported by the Ministry of health, labour and welfare of Japan. Hepatol Res 2014;44:71–90. 24397841 10.1111/hepr.12270

[6] Lindor KD, Bowlus CL, Boyer J, et al Practice guidance from the American association for the study of liver diseases. Hepatology 2018;2019:394–419. 10.1002/hep.30145 30070375

[7] Hirschfield GM, Beuers U, Corpechot C, et al EASL clinical practice guidelines: the diagnosis and management of patients with primary biliary cholangitis. J Hepatol 2017;67:145–72. 10.1016/j.jhep.2017.03.022 28427765

[8] Harms MH, van Buuren HR, Corpechot C, et al Ursodeoxycholic acid therapy and liver transplant-free survival in patients with primary biliary cholangitis. J Hepatol 2019;71:357–65. 10.1016/j.jhep.2019.04.001 30980847

[9] Haboubi H, Shenbagaraj L, Abdul-Sattar A, et al FRI-057-A review of primary biliary cholangitis practice in Wales: time for specialist care. J Hepatol 2019;70:e409–10. 10.1016/S0618-8278(19)30807-2

[10] Lu M, Zhou Y, Haller IV, et al Increasing prevalence of primary biliary cholangitis and reduced mortality with treatment. Clin Gastroenterol Hepatol 2018;16:1342–50. 10.1016/j.cgh.2017.12.033 29277621

[11] Chazouillères O, Wendum D, Serfaty L, et al Primary biliary cirrhosis-autoimmune hepatitis overlap syndrome: clinical features and response to therapy. Hepatology 1998;28:296–301. 10.1002/hep.510280203 9695990

[12] Lammers WJ, van Buuren HR, Hirschfield GM, et al Levels of alkaline phosphatase and bilirubin are surrogate end points of outcomes of patients with primary biliary cirrhosis: an international follow-up study. Gastroenterology 2014;147:1338–49. 10.1053/j.gastro.2014.08.029 25160979

[13] Lanehart RE, Rodriguez de Gil P, Sook Kim E, et al Propensity score analysis and assessment of propensity score approaches using SAS® procedures. SAS Global Forum 2012:5–6.

[14] Robins JM, Hernán Miguel Ángel, Brumback B Marginal structural models and causal inference in epidemiology. Epidemiology 2000;11:550–60. 10.1097/00001648-200009000-00011 10955408

[15] Austin PC The use of propensity score methods with survival or time-to-event outcomes: reporting measures of effect similar to those used in randomized experiments. Stat Med 2014;33:1242–58. 10.1002/sim.5984 24122911 PMC4285179

[16] Altman DG, Andersen PK Calculating the number needed to treat for trials where the outcome is time to an event. BMJ 1999;319:1492–5. 10.1136/bmj.319.7223.1492 10582940 PMC1117211

[17] Lammers WJ, Hirschfield GM, Corpechot C, et al Development and validation of a scoring system to predict outcomes of patients with primary biliary cirrhosis receiving ursodeoxycholic acid therapy. Gastroenterology 2015;149:1804–12. 10.1053/j.gastro.2015.07.061 26261009

[18] Kremers WK Concordance for survival time data: fixed and time-dependent covariates and possible ties in predictor and time. Department of Health Sciences Research and The William J. von Liebig Transplant Center Mayo Clinic, 2007.

[19] Heagerty PJ, Zheng Y Survival model predictive accuracy and ROC curves. Biometrics 2005;61:92–105. 10.1111/j.0006-341X.2005.030814.x 15737082

[20] Parés A, Caballería L, Rodés J Excellent long-term survival in patients with primary biliary cirrhosis and biochemical response to ursodeoxycholic acid. Gastroenterology 2006;130:715–20. 10.1053/j.gastro.2005.12.029 16530513

[21] Carbone M, Mells GF, Pells G, et al Sex and age are determinants of the clinical phenotype of primary biliary cirrhosis and response to ursodeoxycholic acid. Gastroenterology 2013;144:560–9. 10.1053/j.gastro.2012.12.005 23246637

[22] Naylor CD, Chen E, Strauss B Measured enthusiasm: does the method of reporting trial results alter perceptions of therapeutic effectiveness? Ann Intern Med 1992;117:916–21. 10.7326/0003-4819-117-11-916 1443954

[23] Caverly TJ, Prochazka AV, Binswanger IA, et al Confusing relative risk with absolute risk is associated with more Enthusiastic beliefs about the value of cancer screening. Med Decis Making 2014;34:686–92. 10.1177/0272989X14526641 24732049

[24] Bucher HC, Weinbacher M, Gyr K Influence of method of reporting study results on decision of physicians to prescribe drugs to lower cholesterol concentration. BMJ 1994;309:761–4. 10.1136/bmj.309.6957.761 7950558 PMC2541000

[25] Dickson ER, Grambsch PM, Fleming TR, et al Prognosis in primary biliary cirrhosis: model for decision making. Hepatology 1989;10:1–7. 10.1002/hep.1840100102 2737595

[26] Saffioti F, Gurusamy KS, Eusebi LH, et al Pharmacological interventions for primary biliary cholangitis: an attempted network meta-analysis. Cochrane Database Syst Rev 2017;28 10.1002/14651858.CD011648 PMC6464661 28350426

[27] Poupon RE, Poupon R, Balkau B Ursodiol for the long-term treatment of primary biliary cirrhosis. The UDCA-PBC Study Group. N Engl J Med 1994;330:1342–7. 7. FIGURE LEGENDS. 8152446 10.1056/NEJM199405123301903

[28] Lefkowitch JH Liver biopsy assessment in chronic hepatitis. Arch Med Res 2007;38:634–43. 10.1016/j.arcmed.2006.08.005 17613355

[29] ter Borg PCJ, Schalm SW, Hansen BE, et al Prognosis of ursodeoxycholic acid-treated patients with primary biliary cirrhosis. Results of a 10-yr cohort study involving 297 patients. Am J Gastroenterol 2006;101:2044–50. 10.1111/j.1572-0241.2006.00699.x 16848809

